# The role of *Lactobacillus plantarum* in oral health: a review of current studies

**DOI:** 10.1080/20002297.2024.2411815

**Published:** 2024-10-22

**Authors:** Xinyan Huang, Jianhang Bao, Mingzhen Yang, Yingying Li, Youwen Liu, Yuankun Zhai

**Affiliations:** a School of Stomatology, Henan University, Kaifeng, China; b Kaifeng Key Laboratory of Periodontal Tissue Engineering, Kaifeng, China; c Eastman Institute for Oral Health, University of Rochester Medical Center, Rochester, NY, USA; d Orthopedic Department, Luoyang Orthopedic Hospital of Henan Province (Orthopedic Hospital of Henan Province), Zhengzhou, China

**Keywords:** *Lactobacillus plantarum*, caries, periodontal disease, dysbiosis, microbiome, immune response

## Abstract

**Background:**

Oral non-communicable diseases, particularly dental caries and periodontal disease, impose a significant global health burden. The underlying microbial dysbiosis is a prominent factor, driving interest in strategies that promote a balanced oral microbiome. *Lactobacillus plantarum*, a gram-positive lactic acid bacterium known for its adaptability, has gained attention for its potential to enhance oral health. Recent studies have explored the use of *probiotic L. plantarum* in managing dental caries, periodontal disease, and apical periodontitis. However, a comprehensive review on its effects in this context is still lacking.

**Aims:**

This narrative review evaluates current literature on *L. plantarum’s* role in promoting oral health and highlights areas for future research.

**Content:**

In general, the utilization of *L. plantarum* in managing non-communicable *biofilm-dependent* oral diseases is promising, but additional investigations are warranted. Key areas for future study include: exploring its mechanisms of action, identifying optimal strains or strain combinations of *L. plantarum*, determining effective delivery methods and dosages, developing commercial antibacterial agents from *L. plantarum*, and addressing safety considerations related to its use in oral care.

## Introduction

Oral disorders, a significant and pervasive challenge to global public health, have been globally recognized as the primary contributor to all level 3 burden of disease condition based on the comprehensive assessment conducted by The Global Burden of Diseases, Injuries and Risk Factors Study 2017 (GBD2017) [1]. Dental caries, in both deciduous and permanent teeth, and periodontal disease as non-communicable oral disease have affected around 2.8 billion and 796.1 million individuals, respectively, in 2017. Of particular concern is the prevalence of untreated dental caries, which has emerged as a widespread global health issue among human populations. The age-standardized prevalence of untreated dental caries was found to be 29.4% for permanent teeth and 7.8% for primary teeth [1,2].

The dental biofilm stands as a crucial biological determinant shared in the pathogenesis of both caries and periodontal disease [3]. Notably, recent advancements in the understanding of the etiological underpinnings of these diseases have shifted the paradigm towards the concept of microbial dysbiosis, as opposed to the traditional view of infection attributed to individual bacterium [4–6]. These evolving perspectives have catalyzed the development of innovative strategies designed to combat dental caries and periodontal disease, with a primary focus on nurturing a balanced and flourishing oral microbiome. These approaches emphasize the ecological perspective, such as the use of probiotics, in order to rectify the perturbed plaque ecology and promote the establishment and persistence of a symbiotic oral microbiome, ultimately contributing to long-term disease control [7].

Probiotics, defined as ‘live microorganisms which, when administered in adequate amounts, confer a health benefit on the host’ [8]. The overarching goal of probiotics is to prevent or mitigate severe dysbiosis with the aim of establishing a harmonized microbiome that confers beneficial effects upon the host [9,10]. These effects are increasingly acknowledged as capable of positively modulating various facets of human health, also including oral health [11–14].

*Lactobacillus plantarum* is a gram-positive lactic acid bacteria species with probiotic properties [15,16], it exhibits a high degree of heterogeneity, a characteristic underscored by its intricate functional genome and phenotypic variability. All these attributes contribute significantly to its ecological adaptability and metabolic versatility across diverse environmental niches [15–17]. Of particular significance is the aciduric property of *L. plantarum*, which can reduce the membrane fluidity through the activation of adaptive modifications in the fatty acid composition of its plasma membrane when exposed to low pH [18]. Furthermore, this aciduric adaptation involves the up-regulation of proton export facilitated by F0F1‐ATPase [18,19] and the modulation of amino acid metabolism [20].

*L. plantarum*, a probiotic, has demonstrated benefits to human health, especially in the gastrointestinal tract with the function of promoting intestinal integrity, modulating gut microbiome and enhancing mucosal as well as systemic immunity [21–24]. Given the well-established concept of a bidirectional axis connecting the oral cavity and the gastrointestinal tract [25], over the last decade there have been a growing number of studies aimed at deciphering the potential of probiotic *L. plantarum* in the realm of oral health, especially the dysbiosis-related oral disease.

Despite the existence of various narrative or systematic reviews examining the impact of various other probiotic *Lactobacillus* strains on oral health [11,12,26–28], there is a noticeable gap in relation to a dedicated review of the effects of *L. plantarum*. The present narrative review seeks to bridge this gap by offering a comprehensive synthesis of the extant evidence regarding the role of *L. plantarum* in oral health, as well as the underlying mechanisms. Furthermore, we aim to present a succinct overview of areas warranting further investigations, with the ultimate objective of facilitating the future utilization of *L. plantarum* within the realm of oral health.

## *Lactobacillus plantarum*, a Novel Ecological Anti-Caries Candidate in Experimental and Clinical Settings

**Figure 1.**
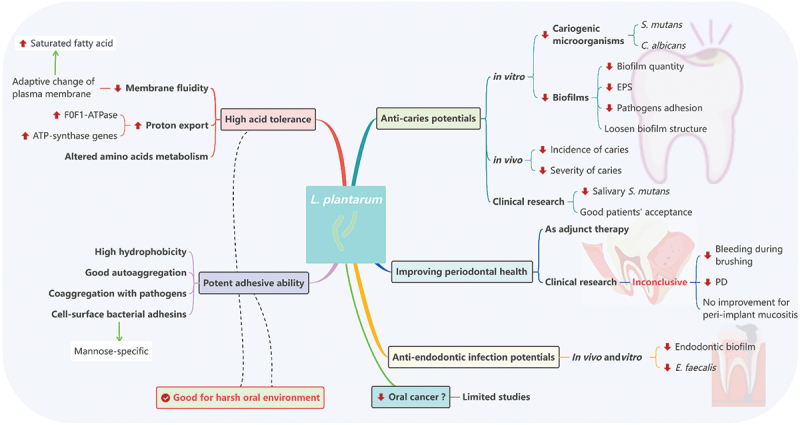
An overview of *L. plantarum* in oral health.

Dental caries, a polymicrobial biofilm-associated disease, is the consequence of dynamic interactions among microorganisms, the host and the host’s diet [29]. These well-established biofilms create a sophisticated micro-environment that enhances biofilm toxicity, impedes the penetration of antimicrobial agents and constrains the buffering capacity of saliva [29,30]. Biofilm resistance and drug tolerance challenge the traditional chemical caries prevention strategy [31]. Probiotic *L. plantarum,* as an ecological approach, has demonstrated its effectiveness in caries control *in vitro, vivo* and in clinical studies [32–46], as shown in Figure 1.

### In vivo and in vitro studies of L. plantarum’s capacities in caries control

Increasing endeavors have been made recently to provide laboratory evidence of utilization of *L. plantarum* in both planktonic and biofilm models. These experiments involved the use of various laboratory or clinically isolated strains (Table 1).

**Table 1.** Experimental evidence of *L. plantarum*’s efficacy in caries control.

*L. plantarum* strain	Form	Study type	Origin of targeted pathogen	Model type	Sampling site	Targeted pathogen	Intervention duration (*in vivo* studies)	Anti-bacterial effect in Planktonic settings	Anti-biofilms effects	Other results
ST-III [33]	WBC	*In vitro*	Clinical isolate	Mono-species planktonic	Saliva	*S. mutans*	/	The ST-III had a strong inhibitory effect on the *S. mutans* with approximately 55.8%-80.0% inhibition rate.	The ST-III significantly reduced the number of *S. mutans, Streptococcus spp* and total bacteria in the mix biofilm cultures.	/
				Multi-species biofilm	Plaque	*S. mutans, Streptococcus spp, Streptococcus sanguinis, Streptococcus sobrinus, Streptococcus salvarius, Porphorymonas gingivalis, Streptococcus oralis*, & *Actinomyces naeslundii*				
ATCC 14,917 [34]	WBC & CFS	*In vitro*	Clinical isolate	Planktonic & mono-species biofilm	Caries dentine	*S. mutans* ATCC 25,175	/	WBC and CFS both showed a significant inhibition zone against *S. mutans*. However, WBC exhibited larger inhibition zone than CFS (*p*<0.05).	1. The 14,917 CFS reduced around 81.0% adherence of *S. mutans* and 25.0% preformed biofilm. 2. The 14,917 changed the EPS matrix structure and quality.	1. After neutralizing the supernatant acidity, the antimicrobial effect was significantly reduced (*p* < 0.01).
										2. Catalase and trypsin treated supernatant didn’t significant influence the antimicrobial effect.
										3. *L. plantarum* stimulate hPBMCs to produce IFN-γ and reduced the IL-10 concentration.
										4. The virulence genes of *S. mutans* were down-regulated in planktonic and biofilms models.
FB-T9 [35]	WBC	*In vitro* & *vivo*	Laboratory strain	Planktonic and mono-species biofilm	/	*S. mutans* ATCC 25,175	70 days	The FB-T9 exhibited good bacteriostatic ability in a plate competition assay.	The FB-T9 significantly reduced the biomass and viability of *S. mutans* biofilms and induced structural damage of biofilm formation.	In a 70-day rat-based *in vivo* experiment:
							Three times a week			1. The FB-T9 significantly reduced the levels of *S. mutans* on the dental surfaces of rats by more than 2 orders of magnitude of the levels in the dental caries model group (*p* < 0.05).
										2. The FB-T9 significantly reduced the caries scores (modified Keyes scoring method) in both the prevention and treatment groups (*p* < 0.05).
CCFM 8724 [36]	WBC	*In vivo*	Laboratory strain & clinical isolate	Planktonic & duo-species biofilm	Caries dentine (*C. albicans*)	*S. mutans* ATCC 25175 & *C. albicans* SJ	40 days	/	/	1. The CCFM8724 in both the treatment and prevention groups could significantly decrease the population of *S. mutans* and *C. albicans* in the rats’ oral cavity (*p* < 0.001), the mineral loss of enamel (*p* < 0.05) and the scores of caries (*p* < 0.05).
							Three times a week			
										2. The CCFM8724 exhibited better effects than chlorhexidine.
108 [37]	CFS	*In vitro*	Clinical isolate	Planktonic & duo-species biofilm	Not mentioned	*S. mutans* UA159 & *C. albicans* SC5314	*/*	The 108 CFS has a significant inhibition on the planktonic mode of growth of both *S. mutans* and *C. albican*s.	The 10^8^ supernatants significantly inhibited the *S. mutans* and *C. albicans* mixed-species biofilm formation and reduced pre-formed mixed-species biofilms with poorly developed biofilm architecture.	The expression of *S. mutans* genes associated with glucosyltransferase activity and *C. albicans* hyphal specific genes (*HWP1, ALS1* and *ALS3*) were down-regulated in the presence of the 10^8^ CFS.
Ln4 [38]	WBC	*In vitro*	Laboratory strain	Mono-species biofilm	/	*S. mutans* KCTC 5124	/	/	1. The Ln4 showed higher antimicrobial activity than *Lactobacillus rhamnosus* GG (LGG).	/
									2. The Ln4-treatment exhibited a lower co-aggregation (58.9%), cell surface hydrophobicity (16.8%) and EPS production rate (73.3%) values, than those of LGG and the negative control.	
									3. The Ln4 effectively inhibited biofilm formation of *S. mutans* KCTC 5124.	
ATCC 8014, ATCC 14,917 [45]	WBC & CFS	*In vitro*	Laboratory strain	Planktonic & duo-species biofilm	/	*S. mutans* UA159 & *C. albicans* SC5314	/	*L. plantarum* demonstrated superior inhibition on the growth of *C. albicans* and *S. mutans.*	*L. plantarum* demonstrated superior disruption of virulent biofilm formation with reduced bacteria and EPS components, and formation of virulent microcolonies structures of *C. albicans-S. mutans* duo-species biofilms.	1. Genes of *S. mutans* and *C. albicans* involved in metabolic pathways were significantly down-regulated.
										2. Genes related to *C. albicans* resistance to antifungal medication (*ERG4*), fungal cell wall chitin remodeling (*CHT2*), and resistance to oxidative stress (*CAT1*) were significantly down-regulated.
										3. *Lactobacillus* genes *plnD, plnG* and *plnN* that contribute to antimicrobial peptide plantaricin production were significantly up-regulated.
										4. *L. plantarum* 14,917 possessed superior inhibition effect than *L. rhamnosus* ATCC 2836, *L. plantarum* ATCC 8014 and *Lactobacillus salivarius* ATCC 11741.
KCTC10887BP [39]	LTA	*In vitro*	Laboratory strain and clinical isolate	Mono-species biofilm	Not mentioned	*S. mutans* KCTC 3065, Ingbritt, OMZ-65, LM-7, KCOM1197 & KCOM1214	/	/	*L. plantarum* LTA inhibited the biofilm formation and aggregation of *S. mutans* without affecting the bacterial growth.	1. Only *L. plantarum* LTA showed a significant inhibition of *S. mutans* biofilm formation among *Lactobacillus sakei*, *Lactobacillus delbrueckii* and *L. rhamnosus* GG.
										2. Notably, *L. plantarum* LTA did not affect the established biofilm.
ATCC 10,012 [46]	CFS	*In vitro*	Laboratory strain	Planktonic & mono-species biofilm	/	*S. mutans* ATCC 25175 & *S. sobrinus* ATCC 33478	/	The 10012 CSC inhibited the growth of *S. mutans* and *S. sobrinus.*	The 10012 CSC significantly reduced biofilm formation by *S. mutans* and *S. sobrinus.*	The 10,012 CSC showed better inhibitory abilities than *Lactobacillus johnsonii* JCM 1022, *L. rhamnosus* ATCC 7469 and *Lactobacillus kefiranofaciens* DD2, DD5 and DD6.
K41 [40]	WBC	*In vitro and vivo*	Not mentioned	Planktonic and mono-species biofilm	/	*S. mutans* UA159	/	The K41 showed the highest inhibitory effect on growth of *S. mutans* and EPS production *in vitro*.	The K41 showed the highest inhibitory effect on the formation of exopolysaccharides (EPS) and biofilm (inhibitory rate: 98.4%) and reduce the network-like structure of biofilm *in vitro*.	1. The coaggregation and autoaggregation rates at 4 h of the K41 were 41.9% and 31.0 %, respectively *in vitro*.
										2. Rats treated with K41 had a significant reduction in the incidence and demineralization degree of dental caries.
LRCC 5193, 5194, 5195 and 5310 [41]	LTA	*In vitro*	Laboratory strain and clinical isolate	Mono-species biofilm	Plaque	*S. mutans* ATCC 25,175 (Lab strain), KCOM 1054, KCOM 1116 & KCOM 1223	/	/	1. The biofilm formations of four strains of *S. mutans* were effectively inhibited by 30μg/ml of LRCC 5310.	ex vivo: The LRCC 5310 LTA dramatically reduced the biofilm formation of clinical isolates of *S. mutans* on human dentin slices.
									2. CFUs from both *Streptococcus gordonii* and *S. mutans* biofilm were dose-dependently attenuated by LRCC 5310 Lp.LTA.	
									3. LTA effect of other *L. plantarum* strains is not conclusive.	
ATCC 8014, ATCC 14,917 [42]	WBC and plantaricin	*In vitro*	Clinical isolates	Planktonic and duo-species biofilm	Not mentioned	*S. mutans* UA159 and *C. albicans* SC5314	/	1. The 14,917 Inhibited the growth of *S. mutans* and *C. albicans* clinical isolates.	The 14,917 inhibited the biofilm formation with a compromised biofilm structure with a significantly smaller microbial and extracellular matrix and a less virulent microcolony structure.	1. The 14917 exhibited better inhibitory effects than *L. salivarius* 11,741 and *L. plantarum* 8014.
								2. The 14,917 reduced the growth of *C. albicans* and inhibited the switching from yeast to the hypha and pseudohypha form.		2. FurTre, one type of plantaricins, produced by *L. plantarum* inhibited the growth of *S. mutans* and *C. albicans.*
										3. The 14,917 had an inhibitory impact on the expression of *S. mutans* and *C. albicans* virulence genes, such as *gtfB, gtfC, atpD, CHT2, ERG4* and *HWP1* in biofilms setting.
ATCC 14,917 [43]	WBC	*In vitro*	Laboratory strain	Planktonic	/	*S. mutans* UA159 and *C. albicans* SC5314	/	1. A dose-dependent inhibition on *C. albicans* and *S. mutans* was observed with the increased dosages of *L. plantarum*.	/	The expression of *C. albicans HWP1, ECE1* and *ERG4* genes and *S. mutans lacC and lacG genes* were significantly downregulated with 10^8^ CFU/mL of *L. plantarum* (*p* < 0.05).
								2. *L. plantarum* at 10^8^ CFU/mL demonstrated the highest antibacterial and antifungal inhibitory effects.		
ATCC 14917 [44]	CFS	*In vitro*	Laboratory strain	mono-species biofilm	/	*S. mutans* UA159	/	/	1. The antibiofilm agent, named 1-1-4-3 is a mixture of lactic acid (LA) and valine.	1. The CFS of *L. plantarum* showed the strongest antibiofilm activity among the tested CFSs of *Lactobacillus casei* ATCC 393, *Lactobacillus gasseri* ATCC 33,323, *Lactobacillus fermentum* ATCC 14,931 and *L. salivarius* ATCC 11741
									2. 1-1-4-3 showed the strongest antibiofilm effect among *L. plantarum* CFS, including reducing the generation of exopolysaccharides and making the biofilm looser and thinner.	2. The catalase treated CFS only show little change regarding anti-biofilm ability.
										3. After the pH was adjusted to 6.5, all tested CFSs lost their antibiofilm ability.

WBC, whole bacteria culture; CFS, cell-free supernatant; LTA, lipoteichoic Acids; EPS, exopolysaccharide.

In summary, *L. plantarum* has demonstrated a potent inhibitory effect on *S. mutans* by reducing bacterial counts and altering the quantity and structure of biofilms. Additionally, the interference with adhesive ability of cariogenic microorganisms to the tooth surface in the presence of *L. plantarum* has been reported by several studies [37,39,41].

Several investigations have undertaken comparative assessments between *L. plantarum* and other *Lactobacilli* strains, such as *L. rhamnosus* GG, *L. sakei*, *L. delbrueckii* and *L. salivarius* 11741, to assess their differential efficacy on cariogenic microorganisms or biofilms [38,39,42,44–46]. These comparative analyses have consistently indicated that *L. plantarum*, in conjunction with its biochemically antimicrobial compounds, exerts the most potent anti-caries effects. These effects encompass the inhibition of pathogenic microorganism proliferation and the prevention of biofilm formation. However, a limited number of studies have explored which specific *L. plantarum* strains possess the most robust impact [40–42,45], resulting in a lack of definitive conclusions.

It is important to note that the majority of these investigations were conducted using the mono-species cariogenic *S. mutans* model or simplified mixed species models that may not fully replicate the intricacies of the oral environment. Additionally, there has been a paucity of *in vivo* studies in this domain [35,36].

### Clinical evidence of L. plantarum’s capacities in caries control

Available clinical information regarding the effectiveness of *L. plantarum* in controlling caries is quite limited, as outlined in Table 2. Gandhi et al. conducted a short-term (14 days) double-blind study involving 60 caries-active children aged 7–10, who were divided into three randomized groups, with one group applying a mucoadhesive patch containing a probiotic blend comprising *L. plantarum* (TSP-Lp1) and *L. rhamnosus*. The study concluded that this probiotic blend exhibited robust antibacterial properties, with a significant reduction in salivary levels of *S. mutans* and its favorable acceptance among patients [47]. Likewise, Lin et al. conducted a clinical trial involving 50 healthy adults who were randomly assigned to two groups. The intervention group received mixed probiotic lozenges containing *L. salivarius subs. salicinius* AP-32, *Lactobacillu paracasei* ET-66 and *L. plantarum* LPL2 for 4 weeks, and found that the administration of mixed probiotics led to a significant reduction in *S. mutans* burden and the inhibition of pathogen growth in saliva [48].

**Table 2.** Clinical evidence of *L. plantarum*’s efficacy in oral health.

Study design/Arms	Participants	*L. plantarum* strain	N (n: n: n)	Delivery vehicle/Dose	Duration/Frequency	Measurements	Conclusion	Reference
RCT/3 arms including I: cinnamon patch, II: probiotic patch and III: control patch (placebo)	Caries-active children aged 7–10 with deft/DMFT score 3 and <5	*L. plantarum* (TSP-Lp1) and *L. rhamnosus*	60 (20: 20: 20)	Mucoadhesive patch/10 mg (5 × 10^10^ for each strain)	14 days/Two patches a day	1. Salivary *S. mutans* CFU/ml count 2. Patient compliance of the presence of adverse effects (taste, breath, teeth staining and nausea)	1. The probiotic incorporated patch caused a highly significant reduction in salivary *S. mutans*. 2. The probiotic patch had good patient acceptance.	Gandhi et al. [47]
RCT/2 arms. I: placebo, and II: viable probiotic lozenge	Healthy non-smoking adults aged 20–40	*L. salivarius subs*. *salicinius* AP-32, *L. paracasei* ET-66 and *L. plantarum* LPL28.	50 (25: 25)	Lozenge/1 g (10^9^ CFU/g totally)	4 weeks/Three lozenges a day	1. Salivary microbiota change 2. Salivary IgA 3. Salivary *S. mutans* abundance 4. Self-reported oral and systemic health improvement	The mixed probiotic lozenge effectively: (1) reduced the number of *S. mutans* in oral cavity, (2) improved the oral microbial flora, (3) increased the performance of IgA antibodies in saliva, (4) decreased oral infections, and improved oral health, including reduced teeth bleeding while brush, (5) attenuated intestinal symptoms.	Lin et al. [48]
RCT/3 arms including I: fluoride, II: probiotic and III: control (placebo)	Healthy fixed orthodontic patients aged 12–30	*L. plantarum* (specific strain was not mentioned)	38 (12: 13: 13)	Mouthwash/10^8^ CFU/each container	2 weeks/Two rinsing a day	Number of *S. mutans* present in dental plaque	The experimental probiotic mouthrinse in use did not show any advantage in terms of controlling *S. mutans*, compared to placebo.	Dadgar et al. [49]
RCT/2 arms including I: placebo, and II: heat-killed (HK) probiotic	Patients with periodontitis undergoing supportive periodontal therapy (SPT)	*L. plantarum* L-137	36 (17: 19)	Capsule/10 mg	12 weeks/One capsule a day	1. Plaque index (PI) 2. Gingival index (GI) 3. Bleeding on probing (BOP) 4. Probing depth (PD)	Daily HK L-137 intake can decrease the depth of periodontal pockets in patients undergoing supportive periodontal therapy.	Iwasaki K et al. [50]
Cross-over RCT/2 arms including I: placebo preparation, and II: home-based administration of probiotics	Systemically periodontally healthy patients rehabilitated with a single, implant-supported unit	*L. plantarum* and *Lactobacillus brevis*	12	Tablet/NA	6 weeks/One tablet a day	1. The number of sites with bleeding on probing (BoP+) 2. Modified plaque index (mPI)	The adjunctive use of probiotics did not significantly enhance the clinical outcomes of professionally administered plaque removal and photodynamic therapy for patients with peri-implant mucositis.	Mongardini C et al. [51]
RCT/2 arms including I: placebo, and II: probiotic gel and lozenges	Individuals with advanced periodontitis seeking periodontal treatment	*L. plantarum* and *L. brevis*	40 (20: 20)	Gel/6.0 × 10^9^ CFU/ml of *L. brevis* and 6.0 × 10^9^ CFU/ml of *L. plantarum* and lozenge/1.2 × 10^9^ CFU/ml of each strain	3 months/One lozenges a day	1. The number of diseased sites (DS: PD > 4 mm + BOP) 2. The number of sites with gingival bleeding	The adjunctive use of probiotics in treating chronic periodontitis results in increased odds for healing of gingival bleeding but reduction of the odds for healing of diseased sites when compared with SRP + placebo; therefore, its use is unfounded.	Pudgar et al. [52]
RCT/2 arms including I: placebo, and II: probiotic tablets	Gingivitis non-smoking patients aged 18–55	*L. plantarum* CECT 7481, *L. brevis* CECT 7480 and *Pediococcus. acidilactici* CECT 8633	59 (30:29)	Tablet/1.00 × 10^3^ CFU/ml of each strain	6 weeks/two tablets a day	1. Gingival index (GI) 2. Relative abundance of 6 periodontal pathogens	The use of probiotic tablets did not lead to significant changes in mean GI. The number of sites with severe inflammation was significantly reduced. The adjunctive use of this probiotic promoted a significant microbiological impact.	Montero et al. [53]

RCT, randomized controlled trial; N (n: n: n), number of the sample size (number of participants in arm I: number of participants in arm II: number of participants in arm III); deft, decayed, extracted due to caries and filled deciduous teeth; DMFT, decayed, missing due to caries and filled permanent teeth.

However, it is worth noting that another randomized, placebo-controlled clinical trial conducted by Dadgar et al., employing the mouthrinse as the delivery vehicle for *L. plantarum*, yielded a contradictory result. They observed that the *L. plantarum* probiotic mouthwash was ineffective in reduction of *S. mutans* levels in dental plaque. The authors acknowledged that variations in *S. mutans* carriage among different groups at baseline and the vehicle form of the mouthwash might have contributed to these unexpected results. This is particularly pertinent considering the limited duration that mouthwash remains in the oral cavity, which may impede the activation of probiotics [49].

In summary, the limited number of clinical studies and the divergent outcomes at present underscore the need for further research efforts. These efforts should particularly focus on determining the optimal *L. plantarum* strain, the most effective concentration, the appropriate duration of administration and the mode of delivery to maximize the potential of *L. plantarum* in caries management.

### Mechanisms network of L. plantarum’s anti-caries abilities

**Figure 2.**
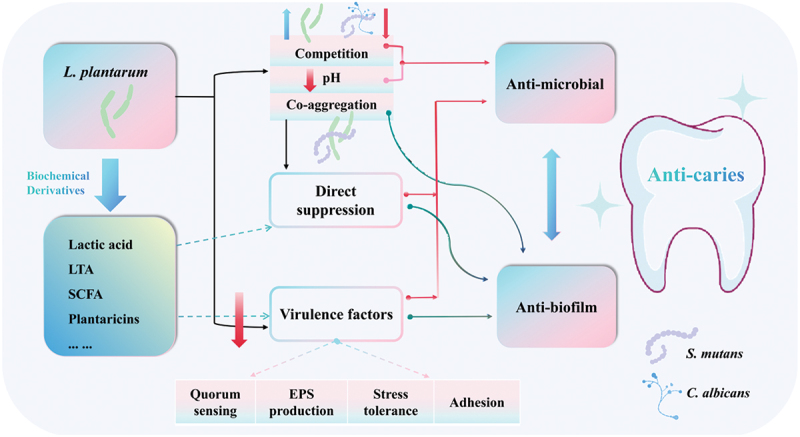
Network of *L. plantarum*‘s anti-caries mechanisms. LTA, lipoteichoic acid; SCFA, short chain fatty acid; EPS, exopolysaccharide.

The potential mechanisms underlying the anti-caries effects of *L. plantarum* have been summarized in Figure 2. Inter-species competition and co-aggregation with cariogenic microorganisms [32,38,40] directly impede the growth and establishment of cariogenic pathogens on teeth surface. As shown in the previous study, *L. plantarum* K41 exhibited an increased co-aggregation with *S. mutans* during the first 4 h and reached 33.6%–44.0% co-aggregation rate at 4 h [40].

The production of antimicrobial substances constitutes a pivotal facet of *L. plantarum*‘s anti-caries potential [34,35,37,39,41,42,44,45]. These antimicrobial substances encompass a spectrum of compounds, ranging from low-molecular-mass compounds such as lactic acid, short chain fatty acid and hydrogen peroxide, to high-molecular-mass compounds known as bacteriocins-peptides that are synthesized within ribosomes and possess antibacterial activity [54]. While lactic acid has been identified as one of the primary antibiofilm agents in caries setting [44], the role of hydrogen peroxide in the anti-caries properties of *L. plantarum* remains contentious. Some studies have suggested that hydrogen peroxide may not be the primary ingredient responsible for the anti-caries properties of *L. plantarum*, as the presence of catalase did not affect the anti-caries effect of *L. plantarum* [34,44]. Currently, the specific substances responsible for the highest anti-caries potency of *L. plantarum* strains have yet to be fully elucidated. Further research is warranted to explore the strain-specific chemical structures and regulatory effects on *L. plantarum*‘s anti-caries properties, with a particular focus on the role of environmental pH. Liang et al. and Wasfi et al. observed a decline or cessation of the antibacterial or antibiofilm properties upon neutralization of the supernatant acidity [34,44].

*L. plantarum* possesses the capacity to influence virulence factors of cariogenic microorganisms, including quorum sensing, biofilm formation and stress tolerance. This influence is evidenced by the suppression of key genes associated with quorum sensing, stress survival, biofilm formation and extracellular polysaccharide (EPS) production, such as *gtfB, gtfC, gtfD* and *sacB* in *S. mutans* [34,37,42,43,45,55]. Furthermore, *L. plantarum* can disrupt the virulence genes of *Candida albicans*, a cariogenic fungus that symbiotically interacts with *S. mutans* and exacerbates biofilm toxicity [56–58]. The *HWP1*, involving in hypha formation, *ERG4*, involving in resistance to antifungal medication, and *ALS* family genes of adhesins, were down-regulated in the presence of *L. plantarum* [37,42,43,59]. There exist inter-strain variations in this capacity. It is worth mentioning that there exists a divergence regarding the expression of *atpD* gene, associated with the acid stress tolerance, between planktonic and biofilm *S. mutans*. Unlike the down-regulation of *atpD* gene observed in biofilm *S. mutans*, several studies have documented the up-regulation of this gene in planktonic *S. mutans* [34,45,59]. These findings suggest that the suppressive effects of *L. plantarum* on *S. mutans* are not characterized by immediate eradication. Instead, they provide an opportunity for *S. mutans* to adapt to the significant decrease in pH caused by *L. plantarum* therapy. This observation aligns with the ecological approach to caries, which emphasizes modulation rather than elimination of endogenous cariogenic microorganisms.

Concerns have arisen regarding the colonization capacity of *L. plantarum* in the oral cavity, particularly under conditions of saliva flow and other harsh oral conditions. Such concerns have led experts to question the feasibility of using *L. plantarum* in this context. Although an *in vitro* study conducted by Jia et al. showed a low adhesive rate of *L. plantarum* AR113 on salivary-coated hydroxyapatite (7.0%) [60], this strain displayed favorable characteristics, including significant autoaggregation, reaching 38.8% within 5 h, and robust adhesion to epithelial cells with a highly uniform distribution across the cellular surface. Furthermore, the coaggregation capacity with *S. mutans* of *L. plantarum* has been certified by several previous *in vitro* studies [32,38,40]. And a study conducted by Zhang et al. revealed that *L. plantarum* possesses a remarkable ability to successfully establish colonization within the oral cavity of rats even within a short administration duration of 5 days [35].

The current understanding of the intricate anti-caries mechanisms of *L. plantarum* remains incomplete, particularly given the complex heterogeneity and various phenotypes of *L. plantarum* strains. Nevertheless, it is evident that the aforementioned mechanisms exhibit a significant degree of overlap and integration. They form a network-like connectivity rather than adhering to a strictly linear relationship. For instance, the Lipoteichoic acid (LTA), a *Lactobacillus*-derived molecule, has been recognized as an antimicrobial substance of *L. plantarum* against biofilm formation of cariogenic microorganisms directly [39,41], with ability to compete and interfere in the binding of *S. mutans* to teeth surface and suppress sucrose deposition [45]. Furthermore, LTA appears to have anti-inflammatory properties, including the attenuating the IL-8 production induced by Pam2CSK4, a synthetic bacterial lipopeptide [61]. Therefore, further investigations are warranted to elucidate these complex interactions fully.

## L. plantarum, a promising adjunct for anti-periodontal disease with favorable attributes and experimental evidence

Periodontal disease is characterized as an irreversible chronic inflammatory disease involving connective tissue destruction, vascular proliferation and alveolar bone destruction induced by immune cells response [62]. The Polymicrobial Synergy and Dysbiosis (PSD) model has been proposed as a conceptual framework for this inflammatory disease [63,64]. In this model, the transformation of host immunity, driven by dysbiotic microbial communities with increased virulence, can further impair immune surveillance and promote an overall inflammatory response [65–67]. The interaction between inflammation and dysbiosis mutually reinforces one another eventually leading to significant changes in the microbial community, and its transition to a pathobiontic state. Prolonged and over-activated inflammation in response to microbial succession, characterized by the domination of proteolytic neutrophils and excessive recruitment of other immune cells, exaggerates tissue and bone degradation [67–70]. Despite ongoing ‘chicken and eggs’ debates on the causative relationship between inflammation and dysbiosis [71], the pursuit of a balanced microbiota emerges as a promising strategy to achieve host-microbe homeostasis and modulate the inflammatory and immune responses in host.

### The potential anti-periodontal disease attributes of L. plantarum

#### Microbiota regulation and anti-microbial mechanisms

Like its anti-caries mechanisms, *L. plantarum* exhibits potential anti-periodontal disease properties through its anti-microbial and microbiota-regulating capacities. This includes enriching the microbiota diversity and suppressing pathogenic microorganisms [72–75]. While the modulation of microbiota by *L. plantarum* has been established in the gastrointestinal tract, its impact on the oral microbiome requires further investigation.

#### Anti-inflammatory and immunomodulatory properties

**Figure 3.**
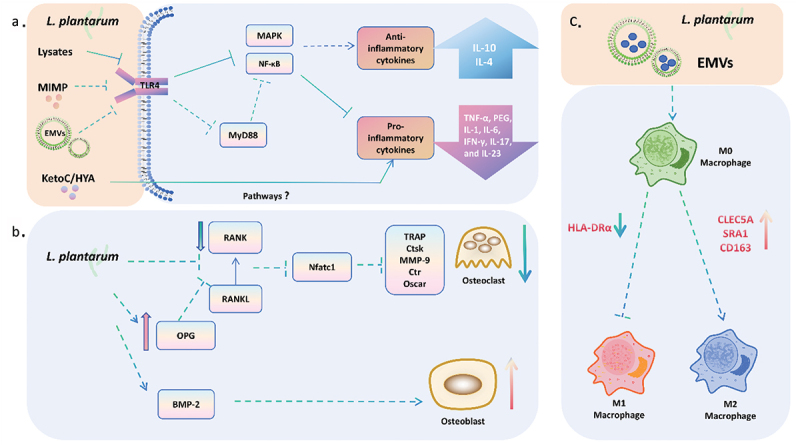
Schematic overview of *L. plantarum*‘s anti-inflammatory, immunomodulatory and osteogenetic modulation. (a) *L. plantarum* and its bioactive components modulate the immune response through TLR4/NF-κB/MAPK pathways, resulting in the reduction of pro-inflammatory cytokines and the release of anti-inflammatory cytokines. (b) *L. plantarum* promotes osteogenesis by modulating osteoblast and osteoclast differentiation. (c) Extracellular membrane vesicles (EMVs) of *L. plantarum* induce M2 macrophages polarization with increased expression of M2-associated cell markers. Solid lines represent evidence from the periodontal domain; dotted lines represent evidence from other systems.

Broadly, *L. plantarum*’s cell-wall components and secreted products interact with the host and mediate the immune homeostasis by regulating cytokine secretion and M2 macrophage polarization (as shown in Figure 3). *L. plantarum* K8 lysates, for instance, reduced TNF-α production in THP-1 cells induced by lipopolysaccharides (LPS) by down-regulating the early signals of mitogen-activated protein kinase (MAPK) and nuclear factor-κB (NF-κB) followed by negative regulators in toll-like receptor (TLR)-mediated signaling *in vitro* [76]. In addition, the nanosized extracellular membrane vesicles (EMVs) of *L. plantarum*, constitutively releasing lipid bilayer-enclosed structures, could reduce pro-inflammatory cytokines levels, including IL-6, IL-1β, IL-2 and TNF-α on gut, by regulating the TLR4-MyD88-NF-κB pathway [77]. Notably, the down-regulation of myeloid differentiation primary response 88 (MyD88) gene expression in this study is of significance, as TLR-MyD88 signaling is pivotal in periodontitis-related osteolysis and MYD88 inhibitors that show preventive effect on jawbone loss in *Porphyromonas gingivalis*-driven periodontitis [78,79].

Furthermore, *L. plantarum*-derived EMVs induce M2b macrophage polarization *in vitro*, as observed by Kim et al. in 2020 [80]. This induction involves biased expression of M2 macrophage-associated cell-surface markers and cytokines (e.g. CLEC5A, SRA1, CD163) and inhibition of the expression of M1 macrophage-associated surface marker HLA-DRα. M2 macrophages, crucial for bone repair and ossification, play a vital role in maintaining a balanced ratio with M1 macrophages in the static periodontal environment [81,82]. An enhanced M2 ratio has been linked to preventing bone loss in periodontitis *in vivo* [83]. Additionally, the micro integral membrane protein (MIMP), a recently identified cell surface protein of *L. plantarum*, exhibits anti-inflammatory properties by modulating inflammatory cytokines. This includes a reduction in pro-inflammatory cytokines such as IFN-γ, IL-17 and IL-23, and an increase in anti-inflammatory cytokines such as IL-4 and IL-10 [84].

### Mitigating bone loss through modulation of osteoblast and osteoclast differentiation

The current studies indicated the potential of *L. plantarum* to ameliorate bone loss through its influence on osteogenesis and anti-osteolysis pathways (Figure 3) [85–88]. *L. plantarum* promotes osteoblast differentiation via the Bone Morphogenetic Proteins (BMP) pathways [85]. Additionally, *L. plantarum* exerts a negative regulatory effect on osteoclastogenesis through Receptor Activator of Nuclear Factor-κB Ligand (RANKL) pathway [85,87]. This inhibition involves the down-regulation of the receptor activator of nuclear factor-κB (RANK) and nuclear factor-activated T cells c1 (Nfatc1) activation, resulting in down-regulated the osteoclast-associated genes, like tartrate-resistant acid phosphatase (TRAP) gene [85,87] *in vitro*. Of note is that TRAP activity is frequently employed as a reliable indication of osteoclast differentiation [89,90]. Moreover, one study suggested that *L. plantarum* can induce osteoprotegerin (OPG), a soluble RANKL decoy receptor that prevents osteoclast formation [88,91].

### L. plantarum’s capacities as adjunct in periodontal disease management in vitro and clinically

Given the intricate interplay of the inflammatory and immune response in periodontal disease [67,68], the well-documented immunomodulatory and inflammation-modulating efficacy of *L. plantarum* in previous studies positions it as a promising adjunct immune-targeted therapy for managing periodontal disease. While *L. plantarum* has shown potential anti-periodontal disease characteristics *in vitro* or *vivo* in the context of other organs or systems, it is crucial to acknowledge that research specifically focused on the periodontal domain, especially underlying mechanism pathways, remains limited currently [55,72,73,92].

Despite limited evidence, available data from two *in vitro* studies confirm the anti-inflammatory properties of *L. plantarum* in the periodontal domain [55,72,92]. Kim. Y et al. [55] revealed that the lysates of *L. plantarum* attenuated *P. gingivalis* LPS-induced phosphorylation of MAPKs and activation of NF-κB in RAW 264.7 cells. Schmitter et al. [92] observed a reduction of inflammatory mediators, PGE2, IL-1, IL-6 and TNF-α, in LPS-stimulated primary monocytes and a decrease in IL-6 release in gingival fibroblasts following the administration of *L. plantarum*. Moreover, the bioactive metabolites 10-oxo-trans-11-oxadecenoic acid (KetoC) and 10-hydroxy-cis-12-octadecenoic acid (HYA), derived from *L. plantarum*, have shown the potential in promoting periodontal homeostasis. These metabolites enhance antioxidant activity through the up-regulation of NRF-2 and HO-1, reduce inflammation by decreasing the levels of pro-inflammatory cytokines TNF-α, IL-6 and IL-1β, and exert antimicrobial effects *in vitro* and *in vivo* [72].

The majority of clinical studies have examined the efficacy of *L. plantarum* as an adjunct therapy for periodontal disease, as presented in Table 2, but these results are inconsistent [48,50–53]. Lin et al. found that consumption of oral lozenges containing mixed probiotics, including *L. plantarum* LPL2, effectively reduced the occurrence of teeth bleeding during brushing [48]. Moreover, a double-blind, placebo-controlled RCT conducted by Iwasaki K et al. used heat-killed *L. plantarum* L-137 in capsules and observed that its administration, in addition to supportive periodontal therapy, and significantly reduced probing depth (*p* < 0.05) at sites with baseline probing depths of ≥4 mm after a 12-week intervention [50]. In contrast, a cross-over RCT conducted by Mongardini C et al. did not find significant improvements in the clinical outcome of mechanical-photodynamic therapy treating experimentally induced peri-implant mucositis with the adjunctive professional and home-based administration of probiotics containing *L. plantarum* and *L. brevis* [51]. Furthermore, the findings of Pudgar et al. indicated an unfavorable impact on periodontal disease, as there was an increased occurrence of residual diseased sites when probiotics containing *L. brevis* and *L. plantarum* strains were used as supplementary therapy alongside scaling and root planning [52].

## L. plantarum, a potential solution for apical periodontitis needing further investigations

Apical periodontitis, an inflammatory disease, arises from bacterial infections originating from biofilms on the root canal wall [93]. The current limitations of intracanal antibacterial agents, including insufficient elimination efficacy, tissue damage and potential allergic reactions, prompt the exploration of probiotics such as *L. plantarum* as promising alternatives. Available evidence indicated that *L. plantarum* and its derivatives hold promise as novel tools for biofilm therapies within the root canal system [72,94–101]. Specifically, *L. plantarum* LTA has exhibited significant suppression of *Enterococcus faecalis*, a microorganism associated with persistent endodontic infections [102], in mono- or mixed-species biofilms, comparable to the routinely used calcium hydroxide [97,99–101]. However, it is crucial to emphasize that research on *L. plantarum*‘s role in addressing apical periodontitis is primarily confined to *in vitro* and *in vivo* studies. Further investigations are warranted to validate these potential therapeutic applications of *L. plantarum* in this specific context.

## Discussion

Our narrative review provides a comprehensive evaluation of the existing literature on the role of *L. plantarum* in promoting oral health and sheds light on possible directions for further research. While the utilization of *L. plantarum* in the oral non-communicable biofilm-dependent disease is encouraging, additional research is needed.

Probiotics have been actively investigated as part of the ecological strategy to maintain oral health [103–106]. Given the established health benefits of *Lactobacillus* and *Bifidobacterium* in the gastrointestinal system, research on probiotics for oral health has concentrated on these probiotic core genera. Specifically, *L. rhamnosus, L. paracasei, L. reuteri* and *L. acidophilus* have undergone extensive investigations, showing promising results in managing both caries and periodontal disease [104–107]. However, research on *L. plantarum* in oral health, a versatile species with a long history of safe use, remains relatively insufficient [15,108]. *L. plantarum* is not only easily accessible, being widely isolated from various niches, including dairy products, vegetables, meat and silage [15,23], but also exhibits ecological adaptability, suitable for harsh oral environment. Moreover, it demonstrated a more potent anti-caries effect compared with other *Lactobacillus* strains, such as *L. rhamnosus GG*, *L. sakei*, *L. delbrueckii* and *L. salivarius* 11741 [38,39,42,44–46]. Therefore, a comprehensive review focusing on *L. plantarum* in oral health is essential.

In the field of caries management, the significant role of *S. mutans* and *C. albicans*, two pivotal cariogenic microorganisms, has been highlighted [109,110], particularly their combined influence in driving the development and potential severity of a dysbiotic/cariogenic oral microbiome [111]. Recent findings indicate a favorable efficacy of *L. plantarum* in controlling cariogenic microorganisms, especially *S. mutans* and *C. albicans*. Of note is that, while one study indicated increased numbers of commensal *S. sanguinis* [33] in multi-species biofilms when co-cultured with *L. plantarum*, a conflicting report suggested *L. plantarum* exerted a non-specific inhibitory effect on cariogenic (*S. mutans*) and health-associated (*S. sanguinis*) species in planktonic settings [112]. These contradictory findings may be due to the different *L. plantarum* strains used and the varying resistance properties of microorganisms in planktonic versus biofilm settings, highlighting the need for caution in the use of probiotics. The role of oral commensal *Streptococci* varies as early colonizers and accessory pathogens dynamically, through mechanisms such as competition, hydrogen peroxide and bacteriocins production, and potentiating pathogenicity [113–115]. Current investigations were conducted lacking a perspective of oral microenvironment and microbiota, which only focus on mono-species models or simplified mixed species models. Hence, it remains inconclusive whether the potential inhibitory effects of *L. plantarum* on oral commensal bacteria are beneficial or not. Recognizing the intricate and dynamic interactions within oral microorganisms [116,117] and the pivotal concept of microbiota transition in oral disease development, there is a growing emphasis on exploring the more representative and complex oral microbiota, instead of individual or a few microbes. For instance, ‘human-derived biofilm models’ [111,118] could be applied to better understand the effects of *L. plantarum* on oral pathogens and commensals.

Moreover, the underlying molecular mechanisms, by which *L. plantarum* and its bioactive compounds regulate bacteria-host homeostasis in the context of periodontal disease, remain poorly understood.

The growing attention towards probiotic *L. plantarum* in oral health has sparked inquiries regarding potential dangers. Concerns revolve around the potential for increased acid production within dental plaque and the occurrence of *Lactobacillus* bacteremia. It is intriguing that there is no significant difference regarding lactic acid levels and overall acid production in dental plaque suspensions in the presence or absence of *L. plantarum* 299 v *in vitro* [119]. Despite the strong acidogenic abilities of *Lactobacillus*, Marttinen et al. reported no change of plaque acidogenicity after consuming other probiotic *Lactobacillus* strains in clinical settings [120]. Moreover, another clinical study noted an increase in salivary pH levels following the consumption of *Lactobacillus* probiotics [121]. Although studies on the safety of *L. plantarum*, particularly regarding acid production, are limited, the observed unexpected change in acidogenicity may be explained by a reduction in cariogenic and acidogenic microorganisms. Additionally, the introduction of *L. plantarum* may promote the activity of other oral lactate-utilizing microorganisms, such as *Veillonella*, through lactate cross-feeding behavior [122]. Lactic acid produced by *L. plantarum* could serve as a carbon source for *Veillonellae* [123,124]. Although the opinions about the effects of oral *Veillonellae* species on oral health and disease are ambivalent, *Veillonellae*’s ability to convert lactic acid to weaker acids and reduce nitrate to nitrite [122,123,125] has been established. In addition, the type of carbohydrate chosen for fermentation impacts the lactic acid production of *L. plantarum*. Combining prebiotic galacto-oligosaccharide with *L. plantarum* has shown effective anti-cariogenic effects with a reduction in acidity in planktonic models, compared to *L. plantarum* alone [59]. Future investigations into the metabolic interaction between *Veillonellae* and *L. plantarum*, as well as exploring the combination of prebiotics and *L. plantarum* as anti-caries strategy within the context of oral microbiota, could be highly intriguing.

*Lactobacillus* bacteremia, while a rare condition, carries a high mortality rate. A review by Kullar R et al. on the topic of probiotics and *Lactobacillus* bacteremia suggests that the use of probiotics can generally be considered safe. However, it is crucial to note that further research is necessary to gain a comprehensive understanding of the potential implications and ensure the safety of probiotic *L. plantarum* usage [126].

Although *L. plantarum* has demonstrated encouraging beneficial effects on oral health both *in vivo* and *in vitro*, multiple considerations need to be addressed to maximize its clinical benefits, besides safety considerations. Utilizing probiotics in the form of multi-strain combinations has been reported to offer a broad spectrum of benefits and potential synergistic effects [127], and changes in the ratios of probiotics may influence the beneficial effects as well as mechanisms pathways [128]. Clinically, evidence supports both disease-specific and strain-specific probiotic chosen strategy [129]. The available studies regarding *L. plantarum* in oral health employed numerous distinct strains. Considering the strain-specific variation of *L. plantarum*, it is important to determine specific combinations and ratios of different *L. plantarum* strains, or combinations of *L. plantarum* with other probiotic genera, for more targeted applications in specific oral disease conditions. In addition, the limited duration of probiotics’ presence in the oral cavity may impede the activation of probiotics. Therefore, factors such as the delivery route, treatment duration, administration frequency and dosage for *L. plantarum* therapy should be carefully considered in clinical practice. Further investigations are essential to promote standardization of therapeutic probiotic application in oral health and maximize the benefits.

## Data Availability

Data sharing is not applicable to this article as no new data were created or analyzed in this study.

## Key messages

The review article presents comprehensive summaries of previous research on the role of *L. plantarum* in promoting oral health. The review aims to provide an overview of the state of *L. plantarum* applications in oral health, to highlight current research gaps and illuminate potential avenues for the further research.
